# The global, regional, and national patterns of change in the burden of bacterial pyoderma from 1990 to 2019 and the forecast for the next decade

**DOI:** 10.1038/s41598-025-85995-z

**Published:** 2025-01-13

**Authors:** Hao Wang, Zihao Bai, Chong Shen, Jiaxi Kou, Yanqing Zhu, Huaxia Xie, Chen Chen, Ran Mo

**Affiliations:** 1 Department of Burns and Plastic Surgery, Nanjing Drum Tower Hospital, Affiliated Hospital of Medical School, Nanjing University, No. 321, Zhongshan Road, Nanjing, 210008 Jiangsu China; 2 Department of Cardiothoracic Surgery, Children’s Hospital of Nanjing Medical University, Nanjing, 210008 China; 3 Nanjing Children’s Hospital, Clinical Teaching Hospital of Medical School, Nanjing University, Nanjing, 210008 China; 4 Department of Burns and Plastic Surgery, Nanjing Drum Tower Hospital Clinical College of Nanjing Medical University, No. 321, Zhongshan Road, Nanjing, 210008 Jiangsu China; 5 Department of Nutrition, Nanjing Drum Tower Hospital, Affiliated Hospital of Medical School, Nanjing University, No. 321, Zhongshan Road, Nanjing, 210008 Jiangsu China

**Keywords:** Pyoderma, Impetigo, Disability-adjusted life-years, Health inequality, Epidemiology, Diseases, Medical research

## Abstract

Pyoderma, commonly known as impetigo, is a bacterial skin infection causing pus formation, prevalent globally, especially in resource-poor areas. It affects both children and adults, including those with conditions like diabetes. Despite its significant impact and economic burden, research on its global epidemiology is limited. This study aims to address this gap by analyzing pyoderma trends from 1990 to 2019 using GBD data. The study aims to analyze global trends in pyoderma epidemiology from 1990 to 2019 using GBD data. Specifically, it investigates Age-Standardized Incidence Rate (ASIR), Mortality Rate (ASMR), and Disability-Adjusted Life Years Rate (ASDR) across 204 countries. Additionally, it provides insights into demographic and socioeconomic factors influencing pyoderma prevalence. Furthermore, it forecasts pyoderma’s development trends for the next decade to inform public health strategies. Data were sourced from the GBD 2019 database, comprising various sources such as censuses, surveys, and registries. Estimates for pyoderma incidence, mortality, and DALYs, along with their 95% uncertainty intervals (UI), were retrieved. The Sociodemographic Index (SDI) was used to assess socioeconomic status, and statistical calculations were performed using the WHO Health Equity Assessment Toolkit and R software (v4.3.2). From 1990 to 2019, the Age-Standardized Incidence Rate (ASIR) and Age-Standardized Mortality Rate (ASMR) of impetigo increased, while the Age-Standardized Disability-Adjusted Life Years Rate (ASDR) declined. Significant global geographical heterogeneity persists, closely associated with the Sociodemographic Index (SDI). Children under 5 and the elderly are particularly at risk, with lower SDI nations bearing higher burdens. Population growth and aging contribute to this rise, with disparities in impetigo trends persisting among countries and regions with varying SDI levels, expected to continue until 2030. Pyoderma exhibits significant heterogeneity across age, gender, and geography, with pronounced disparities evident in underdeveloped regions or countries. Therefore, prioritizing policy formulation and implementing tailored prevention and treatment strategies for high-risk populations are imperative to alleviate the disease burden effectively. Such targeted approaches are crucial in addressing the global impact of pyoderma.

**Supplementary Information:**

The online version contains supplementary material available at 10.1038/s41598-025-85995-z.

## Introduction

Pyoderma, derived from the Greek words pyon meaning “pus” and derma meaning “skin,” refers to a superficial bacterial skin infection commonly known as impetigo. It encompasses any skin disease that is pyogenic, meaning it produces pus. This includes superficial bacterial infections such as impetigo contagiosa, ecthyma, folliculitis, Bockhart’s impetigo, furuncle, carbuncle, and tropical ulcer^1^.

According to statistics, approximately 140 million people worldwide are afflicted with pyoderma, ranking it among the top 50 most common diseases. However, the actual figure may be higher due to limited healthcare resources in resource-poor areas^2^. Pyoderma typically manifests in warm and humid environments, with risk factors including poor sanitation, overcrowding, poverty, and scabies infestation^3^.

Pyoderma is primarily caused by infection with Staphylococcus aureus and Streptococcus pyogenes, and antibiotics constitute the mainstay of treatment^4,5^. Children represent the primary demographic affected, with over 111 million cases worldwide, ranking pyoderma among the three most common skin disorders in children alongside scabies and tinea^6–8^. While prevalence decreases with age, adults with diabetes and other systemic diseases are at increased risk^9^.

Pyoderma imposes a substantial disease burden in resource-poor communities, leading to serious post-infection complications such as cellulitis, septicemia, glomerulonephritis, and rheumatic heart disease, which can be fatal if treatment is delayed^10–12^. The associated costs, including missed school days and the procurement of treatments, further exacerbate the burden on families^13^. Pyoderma and scabies also pose significant financial burdens on individuals, families, and health services, accounting for a notable percentage of visits to primary healthcare centers in tropical countries^6^.

Currently, there is a lack of comprehensive research on global pyoderma and its influencing factors. In order to gain a deeper understanding of the extent of pyoderma as a global health issue, we advocate for enhanced research efforts and policy support to mitigate its negative societal impact. This study utilizes the latest available data from the Global Burden of Disease (GBD) project to analyze the age, gender, and sociodemographic index (SDI) trends in pyoderma Age-Standardized Incidence Rate (ASIR), Age-Standardized Mortality Rate (ASMR), and Age-Standardized Disability-Adjusted Life Years Rate (ASDR) from 1990 to 2019 across 204 countries and territories. Additionally, we forecast the epidemiological trends of pyoderma for the next decade and provide a comprehensive summary of its global incidence patterns.

## Methods

### Data source

The data utilized in this study were obtained from the GBD 2019 database, which can be accessed via the GBD results tool hosted on the Institute for Health Metrics and Evaluation (IHME) website (http://ghdx.healthdata.org/)^14^. The GBD 2019 estimation process combines a range of data sources, such as censuses, household surveys, civil registration and vital statistics systems, disease registries, health service utilization records, air pollution monitoring data, satellite imagery, disease notifications, among others. This extensive data collection is the result of systematic literature reviews, searches on government and international organization websites, analysis of reports, and assessments of primary data sources like population and health surveys. Additionally, the dataset incorporates contributions from collaborators involved in the GBD project^15^.

For this study, estimates of incidence, mortality, and DALYs for pyoderma, along with their corresponding 95% uncertainty intervals (UI), were extracted from the GBD 2019 database. Furthermore, the study utilized the Sociodemographic Index (SDI), which is a composite measure assessing the socio-demographic status of a country or region based on average income, educational attainment, and fertility rates^16^.

### Cross-country inequality analysis

The inequality slope index and the concentration index serve as standardized metrics to assess both absolute and relative gradient inequalities. They are utilized to gauge the disparity in the distribution of pyoderma burden across different countries. The inequality slope index is derived from regression analysis, which correlates a nation’s DALYs rates with its relative position in SDI, determined by the midpoint of the population in a cumulative SDI distribution. Heteroscedasticity is addressed through the utilization of a weighted regression model. Conversely, the concentration index is computed by integrating the area beneath the Lorenz Curve, aligning the cumulative percentage of DALYs with the cumulative distribution of the population sorted by SDI^17^.

### Decomposition analysis

In order to understand the primary drivers behind the fluctuations in pyoderma burden from 1990 to 2019, a decomposition analysis was conducted. This analytical approach aimed to measure the distinct influences of population growth, aging, and epidemiological changes. Each factor’s contribution was evaluated independently while keeping the other two factors constant.

### Statistics

Incidence, mortality and DALYs rates are presented as estimates per 100,000 population, accompanied by their 95% UI. The analyses and visualizations in this study were conducted using the World Health Organization Health Equity Assessment Toolkit along with R software (version 4.3.2).

## Result

### Global burden of pyoderma across 204 countries in 2019

**Fig. 1:**
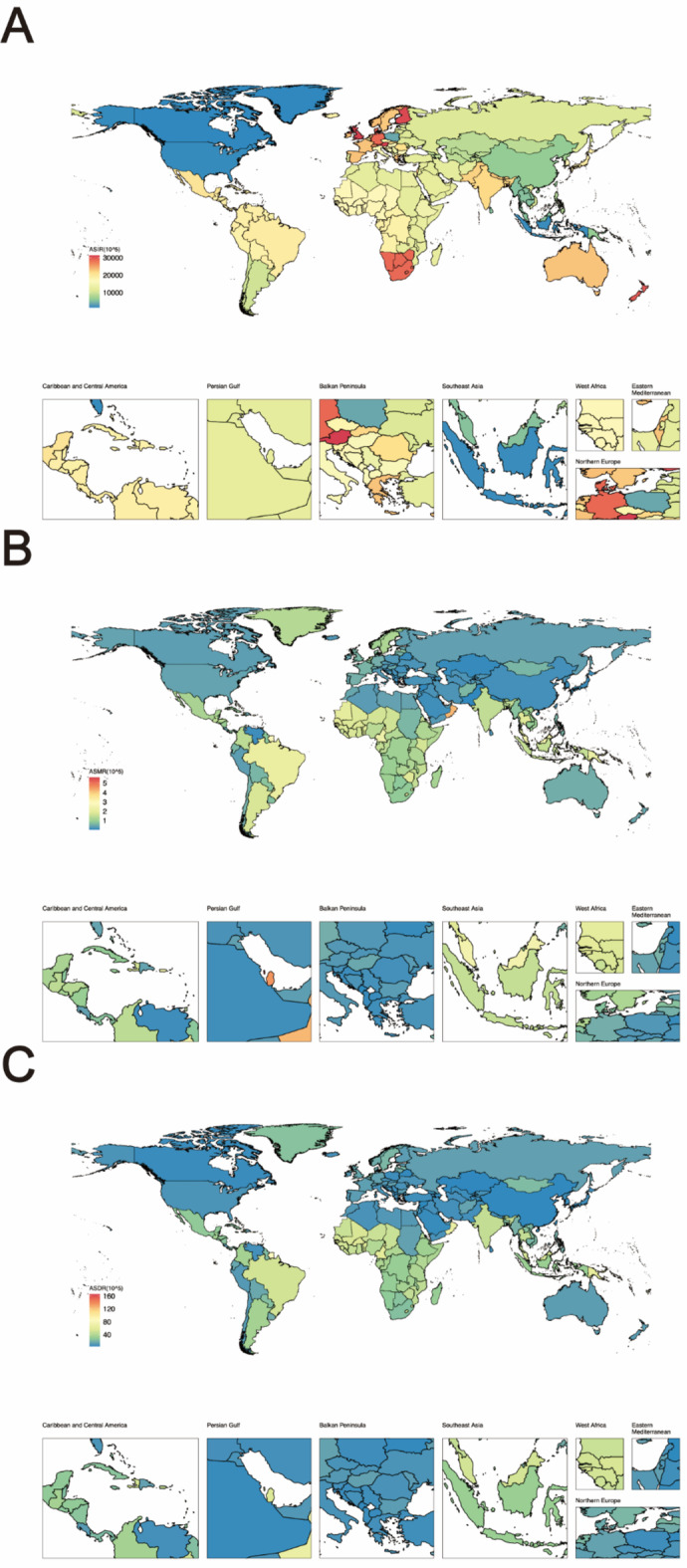
Age-standardized Incidence, Mortality and DALYs rates of pyoderma Globally across 204 countries and territories in 2019. (**A**) Age-Standardized Incidence Rate (ASIR) of pyoderma; (**B**) Age-Standardized Mortality Rate (ASMR) of pyoderma; (**C**) Age-Standardized Death Rate (ASDR) of pyoderma.

In 2019, the Republic of Austria, the United Kingdom of Great Britain and Northern Ireland, and the Republic of Finland exhibited higher ASIR values. Conversely, countries with the lowest ASIR values included Canada, Greenland, and the United States of America (Fig. 1A, Table S1).

Meanwhile, in 2019, American Samoa, the Kingdom of Bahrain, and the State of Qatar had the highest ASMR, while the lowest ASMR was observed in the Islamic Republic of Pakistan, Montenegro, and the Federal Democratic Republic of Nepal (Fig. 1B, Table S2).

American Samoa, Grenada, and the Northern Mariana Islands ranked among the top three countries in ASDR in 2019. In contrast, the Republic of Kazakhstan, the People’s Republic of China, and the Syrian Arab Republic had the lowest incidence rates (Fig. 1C, Table S3).

### Age and sex distribution of pyoderma burden

**Fig. 2:**
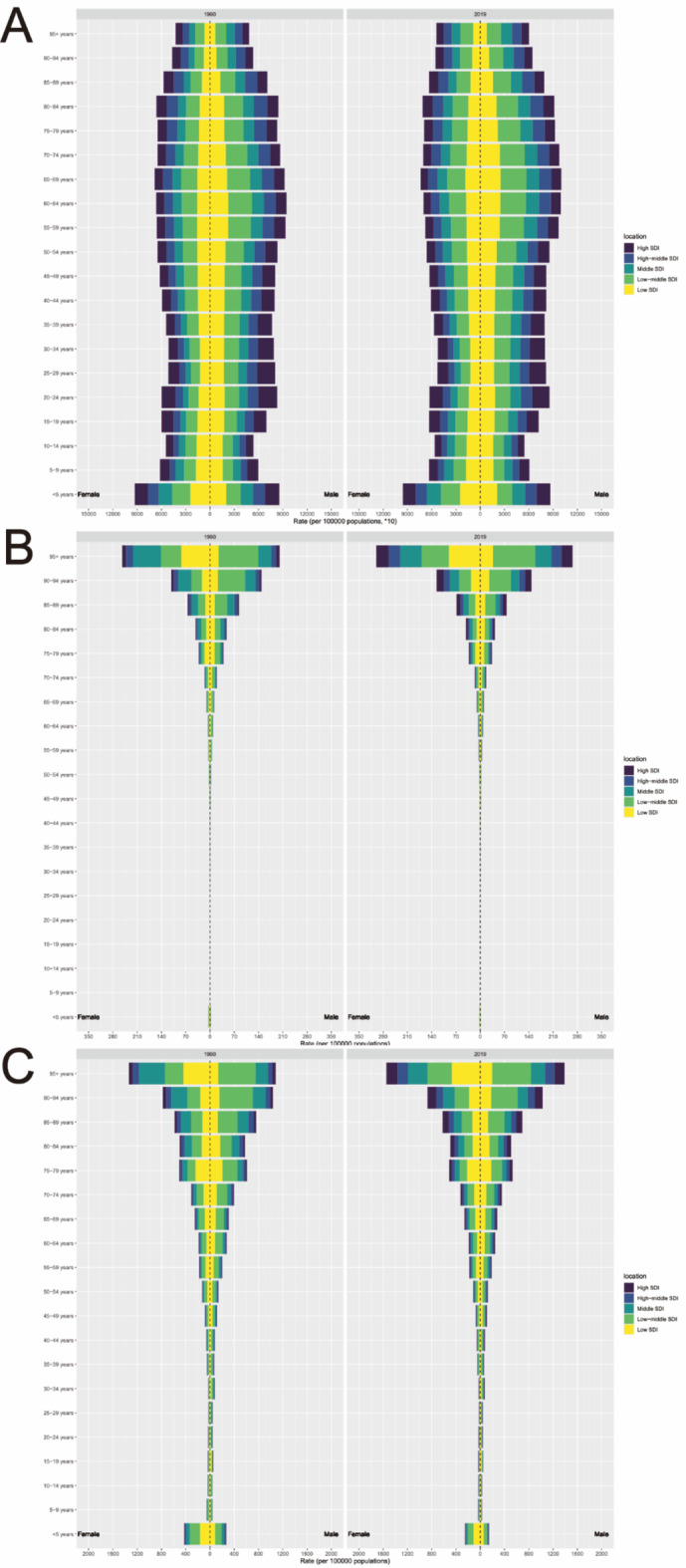
Age and sex distribution of pyoderma in different sociodemographic index (SDI) regions in 1990 and 2019. (**A**) ASIR of pyoderma; (**B**) ASMR of pyoderma; (**C**) ASDR of pyoderma.

In terms of ASIR for pyoderma, there have been no significant changes observed between 1990 and 2019. Children under 5 years old have the highest incidence rates, primarily occurring in regions with middle, low-middle, and low SDI levels. There are no significant differences in incidence rates between genders across all age groups (Fig. 2A).

Regarding ASMR for pyoderma, there have been no notable changes observed between 1990 and 2019. No significant mortality rates were observed in the age group of 5 to 44 years, with mortality rates increasing with age, particularly among patients aged 95 and above. Similar to incidence rates, mortality rates are predominantly concentrated in regions with middle, low-middle, and low SDI levels. In regions with low-middle SDI levels, male patients constitute the majority of mortality cases across all age groups. However, in regions with low SDI levels, females comprise the majority of mortality cases among patients aged 95 and above (Fig. 2B).

As for ASDR for pyoderma, there have been significant changes observed in children under 5 years old from the past to present, showing a noticeable decline thereafter, and increasing after the age of 45. DALYs are similarly concentrated in regions with middle, low-middle, and low SDI levels. In regions with low-middle SDI levels, male patients account for the majority of DALYs across all age groups. However, among patients aged 95 and above in regions with low SDI levels, females constitute the majority of DALYs (Fig. 2C).

### Cross-country social inequalities analysis

Using the Slope Index of Inequality (SII) and Concentration Index, absolute and relative inequalities related to SDI were observed in ASIR, ASMR, and ASDR for pyoderma.

**Fig. 3:**
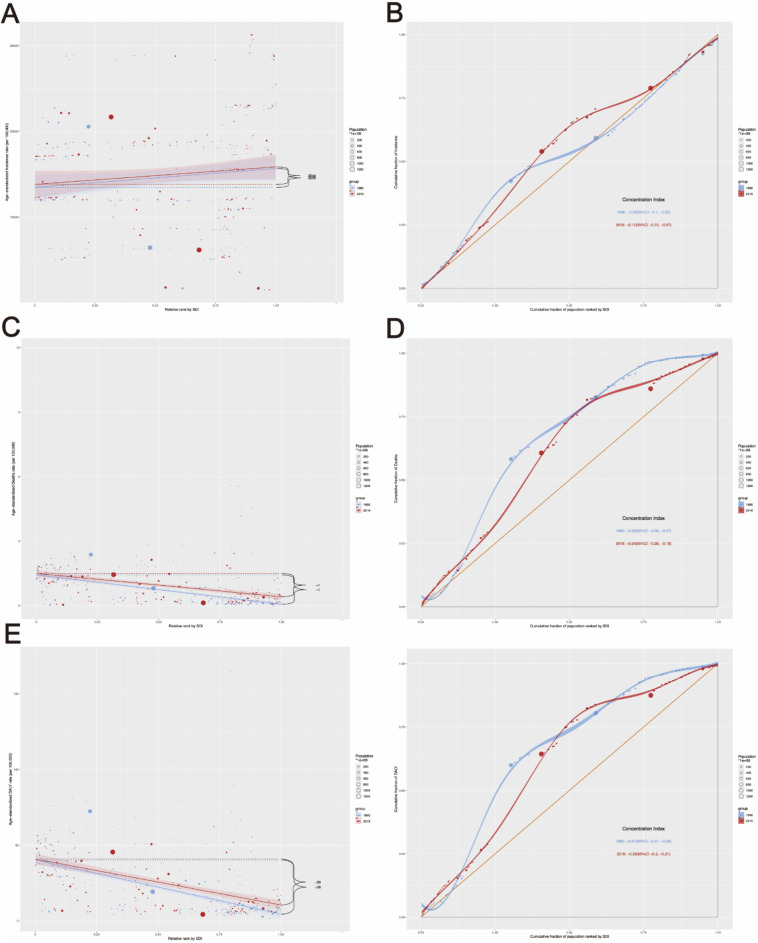
Slope Index of Inequality and concentration curves for the burden of pyoderma from 1990 to 2019. (**A**) Health inequality regression curve for incidence rate; (**B**) Health inequality concentration curve of incidence rate; (**C**) Health inequality regression curve for mortality rate; (**D**) Health inequality concentration curve of mortality rate; (**D**) Health inequality regression curve of DALYs rate; (**D**) Health inequality concentration curve of DALYs rate.

From 1990 to 2019, the SII for the incidence rate of pyoderma decreased from 2205 (95% CI: − 691.16, 5101.96) to 2044 (95% CI: − 886.43, 4974.31). The Concentration Index indicated − 0.06 (95% CI: − 0.10 to − 0.01) in 1990 and − 0.11 (95% CI: − 0.15 to − 0.07) in 2019 (Fig. 3A, B, Table S4, S5).

The SII for mortality rate decreased from − 1.4 (95% CI: − 1.56, − 1.16) to − 1 (95% CI: − 1.35, − 0.83) during the same period. The Concentration Index showed − 0.32 (95% CI: -0.38 to -0.27) in 1990 and − 0.24 (95% CI: − 0.29 to − 0.18) in 2019 (Fig. 3C and D, Table S4 and S5).

For DALYs rates, the SII decreased from − 36 (95% CI: − 40.65, − 30.91) to − 30 (95% CI: − 36.52, − 24.17). Additionally, the Concentration Index was − 0.31 (95% CI: − 0.37, − 0.26) in 1990 and − 0.26 (95% CI: − 0.30 to − 0.21) in 2019 (Fig. 3E and F, Table S4 and S5).

### Drivers of pyoderma epidemiology: aging, population and epidemiologic changes

A decomposition analysis of pyoderma was conducted from three aspects: Incidence, Deaths, and DALYs, to investigate the extent of the impact of Aging, Population, and Epidemiological changes on the epidemiology of pyoderma from 1990 to 2019.

**Fig. 4:**
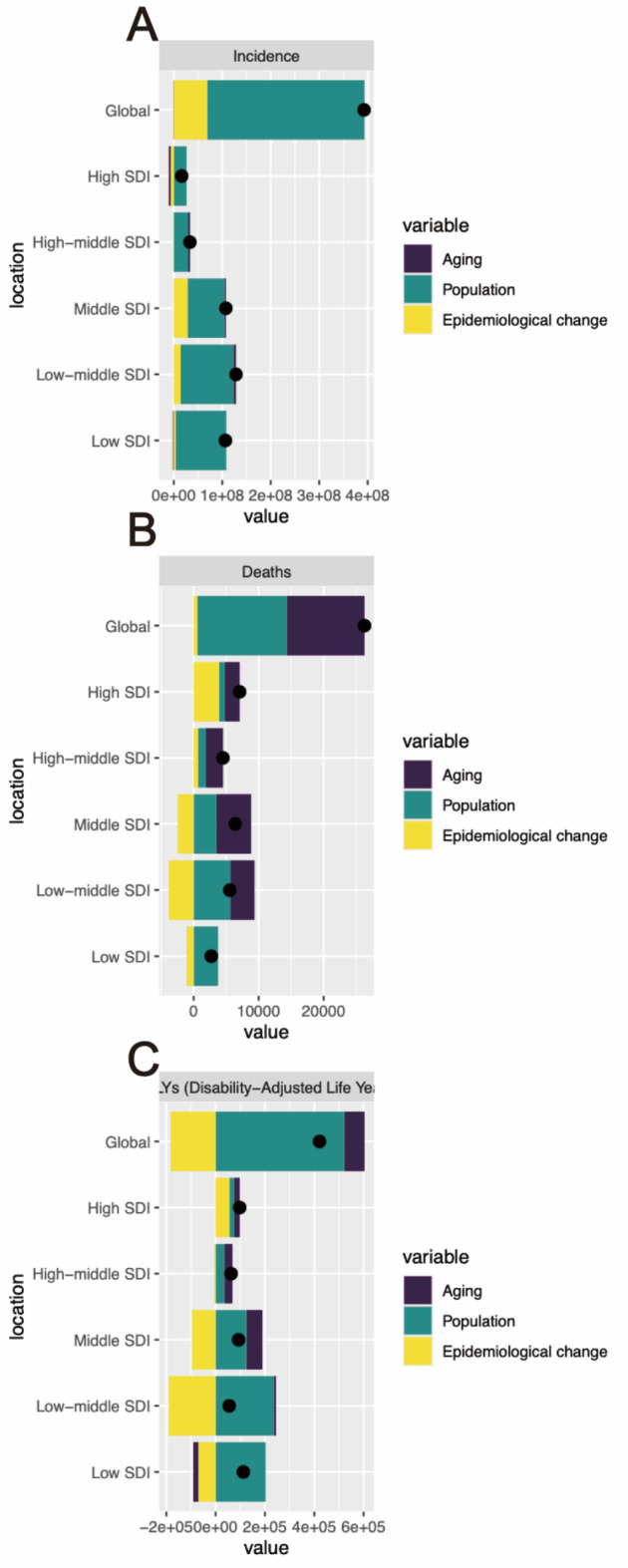
Decomposition analysis of age-standardized incidence, mortality and DALYs rate of pyoderma. (**A**) Decomposition analysis of pyoderma incidence rate; (**B**) Decomposition analysis of pyoderma mortality rate; (**C**) Decomposition analysis of pyoderma DALYs rate.

In terms of incidence rates, a global and SDI-specific upward trend was observed, with the most significant increases observed in regions with middle, low-middle, and low SDI levels (Fig. 4A, Table S6). Globally, the burden of increased incidence rates was primarily attributed to population growth (82.69%), while Aging and Epidemiological changes accounted for a lower proportion (− 0.22%, 17.53%). Population growth was prominent across all SDI regions, while Aging and Epidemiological changes had relatively minor proportions in each SDI region, showing no significant heterogeneity.

Similarly, for deaths rates, a global and SDI-specific upward trend was observed, with the most significant increases observed in regions with middle, low-middle, and high SDI levels (Fig. 4B, Table S6). Globally, the burden of increased mortality rates was primarily attributed to Aging and Population (45.52%, 52.37%), while Epidemiological changes accounted for a lower proportion (2.1%). Aging had a certain proportion in all regions except for low SDI regions, while population growth had a larger proportion in regions with middle, low-middle, and low SDI levels. Notably, Epidemiological changes were positive in regions with high and high-middle SDI levels and negative in regions with middle, low-middle, and low SDI levels. The decomposition analysis of GBD regions showed some heterogeneity.

Regarding DALYs, a global and SDI-specific upward trend was observed, with the most significant increases observed in regions with middle, low-middle, and low SDI levels (Fig. 4C, Table S6). Globally, the burden of increased DALYs was primarily attributed to population growth and Aging (123.9%, 19.4%), while Epidemiological changes were negative (-43.3%). Population growth was prominent in all regions except for high SDI regions. Aging was prominent in high-middle and middle SDI regions and negative in low SDI regions. Epidemiological changes were pronounced in high SDI regions and negative in the rest. The decomposition analysis of GBD regions showed significant heterogeneity.

### Frontier analysis involving SDI and pyoderma burden

Utilizing data from 1990 to 2019 on ASIR, ASMR, ASDR, and SDI, a frontier analysis was conducted. The frontier, depicted in solid black color, represents the optimal scenario of disease burden under a specific SDI level. The distance from the frontier, termed effective difference, signifies the gap between the observed and potentially achievable indicators within a country. Reasonable utilization of a nation or region’s social demographic resources could potentially narrow or eliminate this gap. Blue dots represent countries with low SDI (< 0.5) and low effective difference, while red dots represent countries with high SDI (> 0.85) and high effective difference. Black dots denote the 15 countries with the most significant effective difference.

**Fig. 5:**
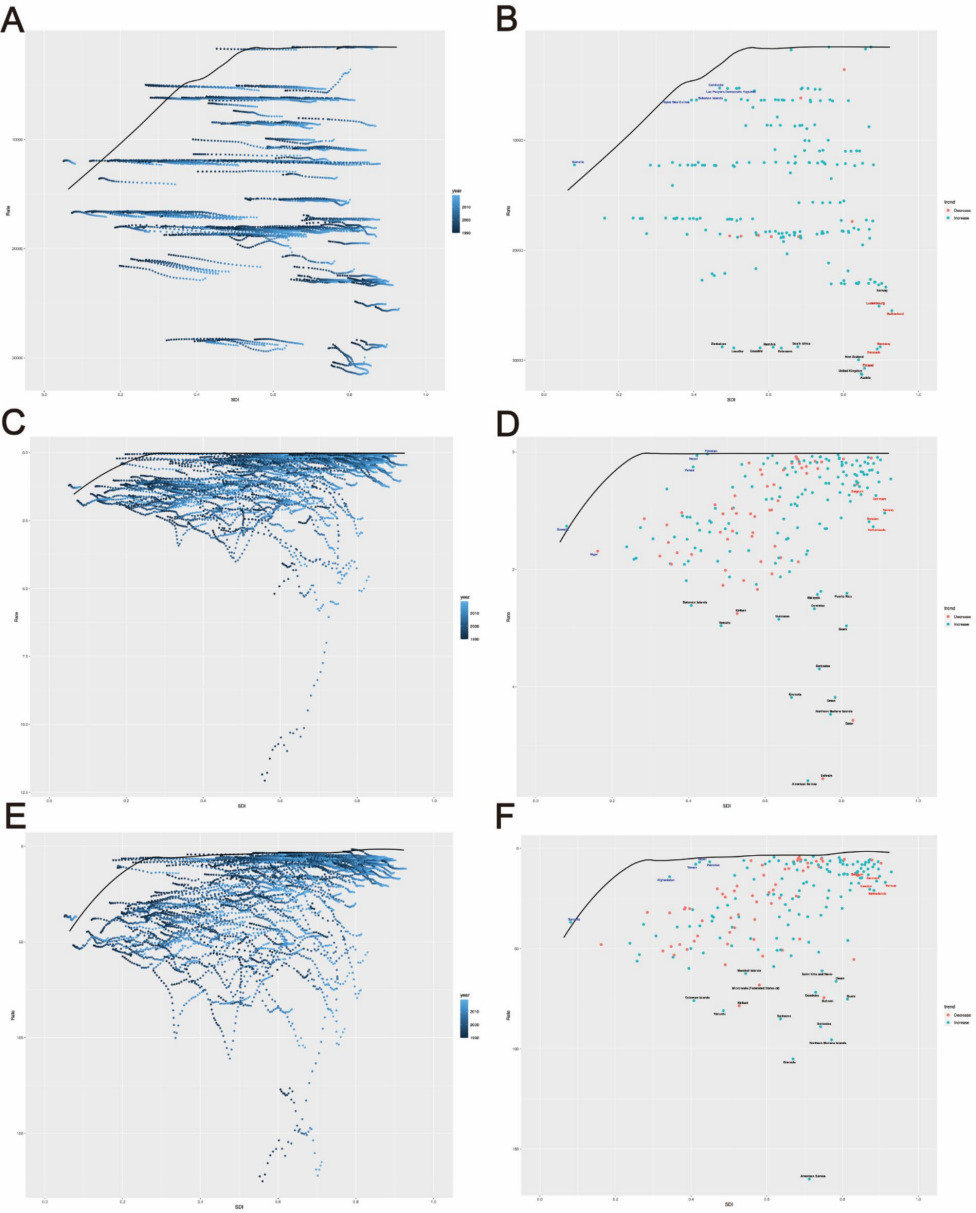
Frontier analysis involving SDI and pyoderma burden. (**A**,** B**) Frontier analysis based on SDI and ASIR from 1990 to 2019; (**C**,** D**) Frontier analysis based on SDI and ASMR in 2019; (**E**,** F**) Frontier analysis based on SDI and ASDR from 1990 to 2019.

In terms of ASIR, countries with low SDI (< 0.5) and low effective difference primarily include Somalia, Solomon Islands, and Papua New Guinea, while countries with high SDI (> 0.85) and high effective difference encompass Finland, Denmark, and Germany. Countries with the most significant effective difference include Austria, the United Kingdom, Finland, New Zealand, and Denmark (Fig. 5A and B, Table S7).

Regarding ASMR, countries with low SDI (< 0.5) and low effective difference predominantly comprise Pakistan, Nepal, Somalia, Yemen, and Niger, whereas countries with high SDI (> 0.85) and high effective difference include the Netherlands, Sweden, and Norway. The countries with the most significant effective difference consist of American Samoa, Bahrain, and Qatar (Fig. 5C and D, Table S8).

As for ASDR, countries with low SDI (< 0.5) and low effective difference mainly consist of Somalia, Pakistan, and Nepal, while countries with high SDI (> 0.85) and high effective difference encompass the Netherlands, Sweden, and Norway. The countries with the most significant effective difference include American Samoa, Grenada, and the Northern Mariana Islands (Fig. 5E, F, Table S9).

### Joinpoint regression analysis of pyoderma

Next, we utilized Joinpoint regression models to analyze the temporal trends of global pyoderma-related ASIR, ASMR, and ASDR.

**Fig. 6:**
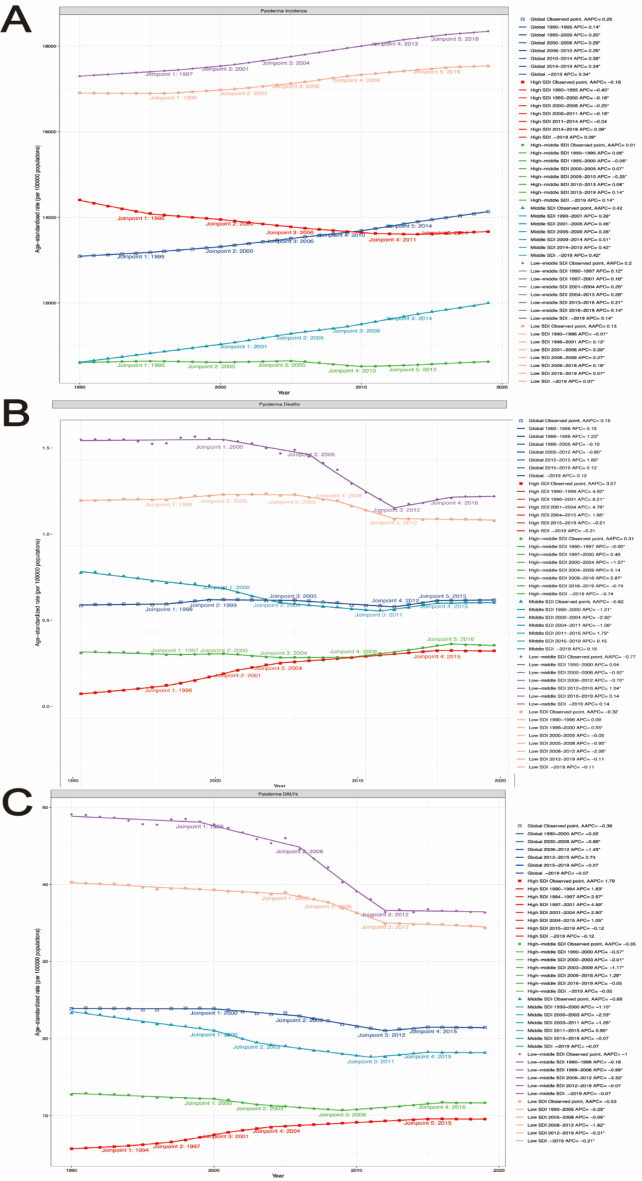
Joinpoint regression analysis of the ASIR, ASMR and ASDR for pyoderma in Global from 1990 to 2019. (**A**) ASIR for pyoderma; (**B**) ASMR for pyoderma; (**C**) ASDR for pyoderma.

On a global scale, pyoderma ASIR demonstrated an increasing trend overall (AAPC 0.26, 95% CI: 0.26 to 0.27), except for a decreasing trend observed in high SDI countries (AAPC − 0.18, 95% CI: − 0.19 to − 0.17), with all other regions showing an increase in ASIR (Fig. 6A, Table S10).

Global pyoderma ASMR exhibited an increasing trend overall (AAPC 0.15, 95% CI: − 0.04 to 0.34), with high SDI regions (AAPC 3.57, 95% CI: 3.44 to 3.70) and high-middle SDI regions (AAPC 0.31, 95% CI: − 0.05 to 0.68) showing an increase in mortality rates, while moderate SDI (AAPC − 0.82, 95% CI: − 0.98 to − 0.65), low-middle SDI (AAPC − 0.77, 95% CI: − 0.99 to − 0.54), and low SDI regions (AAPC − 0.32, 95% CI: − 0.46 to -0.19) exhibited a decrease in ASMR (Fig. 6B, Table S10).

Regarding ASDR, global pyoderma DALY rates demonstrated an overall decrease (AAPC − 0.38, 95% CI: − 0.53 to − 0.22), with the exception of an increasing trend observed in high SDI regions (AAPC 1.79, 95% CI: 1.68 to 1.91), while all other regions showed a decrease (Fig. 6C, Table S10).

### Prediction of pyoderma burden in worldwide from 2020 to 2030

**Fig. 7:**
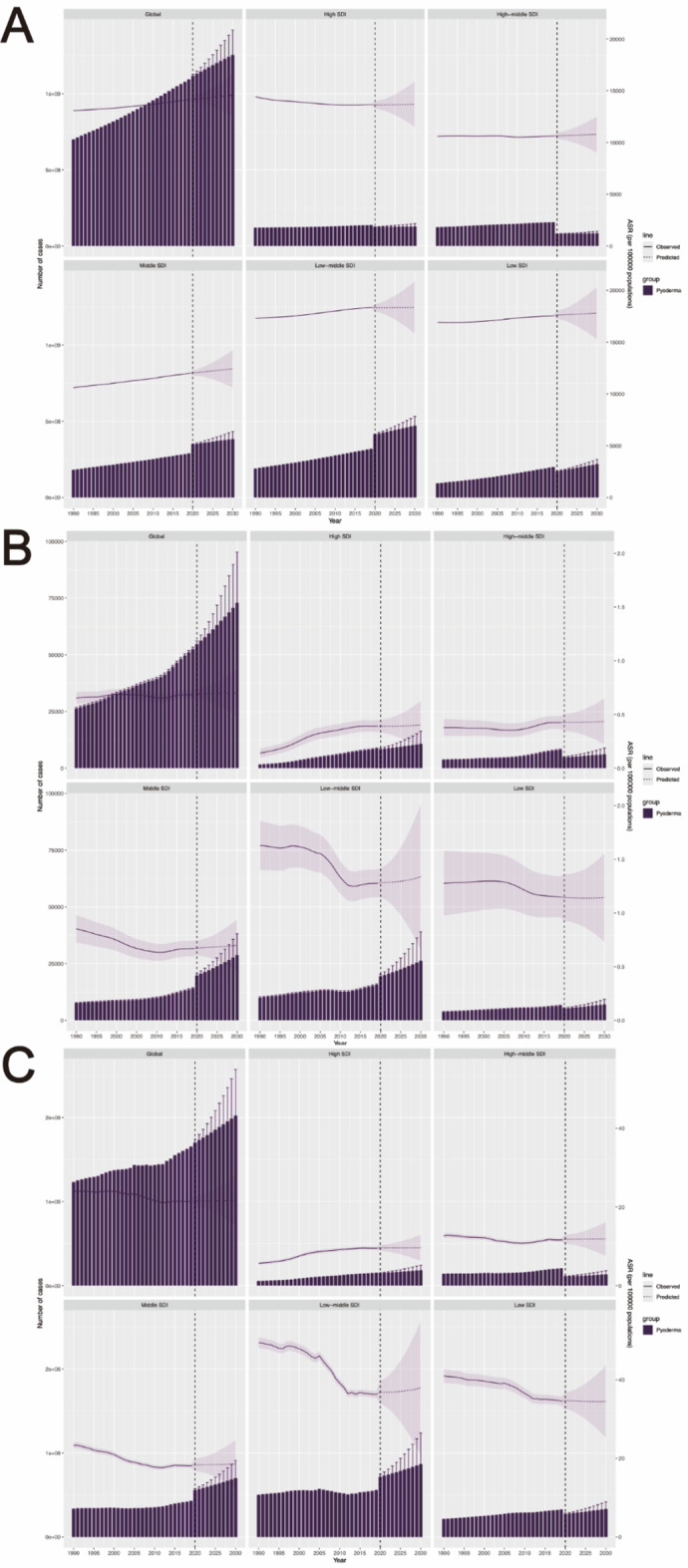
Predictions of pyoderma globally from 2020 to 2030. (**A**) Incidence number and rate; (**B**) Mortality number and rate; (**C**) DALYs number and rate.

Finally, we used Bayesian age-period-cohort (BAPC) to forecast the incidence, mortality, and DALYs rates of global pyoderma from 2019 to 2030. We found that over the next decade, there is a projected slight increase in the incidence, mortality, and DALYs rates of pyoderma. Specifically, for the incidence rate, it is expected to increase from 14,136 cases per 100,000 individuals in 2019 to 14,568 cases per 100,000 individuals in 2030, representing a 3% increase (Fig. 7A, Table S11). Regarding the mortality rate, it is projected to increase from 0.69 cases per 100,000 individuals in 2019 to 0.73 cases per 100,000 individuals in 2030, reflecting a 6% increase (Fig. 7B, Table S11). As for the DALYs rate, it is forecasted to rise from 21.43 cases per 100,000 individuals in 2019 to 21.65 cases per 100,000 individuals in 2030, indicating a 1% increase (Fig. 7C, Table S11).

## Discussion

In this study, we present a comprehensive and current analysis by consolidating pyoderma-related data spanning from GBD 1990 to 2019. Our aim is to elucidate the contemporary landscape of pyoderma burden, delineating its prevalence across age groups, genders, and geographical regions. Specifically, we scrutinize the notable disparities in pyoderma burden across different SDI level, examining metrics such as ASIR, ASMR, and ASDR. Additionally, we endeavor to forecast the trajectory of pyoderma incidence over the next decade, thereby offering insights into the divergent pyoderma profiles across 204 nations and territories.

In 2019, ASIR, ASMR, and ASDR for pyoderma exhibited significant disparities among countries worldwide. ASMR and ASDR were notably elevated in regions with lower SDI, indicative of a heightened burden of pyoderma. This phenomenon can be attributed to a confluence of factors including inadequate sanitation, limited healthcare resources, and socio-economic challenges prevalent in resource-deprived areas^2^. The risk factors for pyoderma also include inflammatory bowel disease, metabolic risk factors, alcohol consumption, and other skin diseases, all of which have a significant impact on the prevalence and progression of the disease^18^. These comorbidities contribute to an increased susceptibility to infections and may exacerbate the severity of pyoderma in affected individuals. Additionally, the microbiology, risk factors, diagnostic methodologies, and accessibility of treatment exhibit considerable variation based on the contextual setting^19^. It is noteworthy that most studies underscore a more pronounced prevalence of pyoderma in tropical and subtropical developing nations. Conversely, there has been relatively less emphasis on pyoderma in high SDI countries in Europe, likely stemming from a diminished prevalence^10,19^. However, our research reveals that even though the aggregate ASMR coupled with the ASDR indicates a higher burden of pyoderma in low SDI regions, certain high SDI countries in Europe (such as the Republic of Austria, the United Kingdom of Great Britain and Northern Ireland, and the Republic of Finland) demonstrate a higher ASIR for pyoderma. Partial studies corroborate this observation^20,21^. This uptick has been linked to the widespread emergence of a specific impetigo strain in Europe, recognized as the epidemic European fusidic acid-resistant impetigo clone (EEFIC), falling under clonal complex (CC) 121 and exhibiting notable resistance to fusidic acid at a low level^22–26^. This finding underscores the imperative to not confine pyoderma to specific regions but to escalate global awareness and treatment efforts, emphasizing the overarching challenge of pyoderma as a public health concern.

By employing a Joinpoint regression model for pyoderma, our analysis reveals a prevailing upward trend in both incidence and mortality rates globally, juxtaposed with an overall decreasing trend in DALYs rates. This suggests a nuanced scenario wherein while the incidence and mortality rates of pyoderma are on the rise in certain regions, advancements in medical care, treatment modalities, and preventive measures contribute to a diminishing proportion of pyoderma’s overall impact on population health. Nonetheless, the absolute number of individuals affected by pyoderma continues to escalate. Through the application of the SII and the Concentration Index (CI), our study underscores the persistent global health inequalities associated with pyoderma, closely tethered to SDI. This underscores the pivotal role of social determinants such as healthcare access, economic prosperity, and educational attainment in pyoderma prevention and management.

The often-overlooked health ramifications of scabies and pyoderma in resource-scarce regions are gradually garnering attention, exemplified by the establishment of the International Scabies Control Alliance and the inclusion of scabies in the World Health Organization’s roster of neglected tropical diseases in 2013^27^. Metabolic risk factors, particularly those related to lifestyle and socio-economic conditions, may also contribute to the elevated incidence of pyoderma in lower SDI countries. As highlighted in recent studies on cardiometabolic conditions, such as the increasing burden of obesity, type 2 diabetes, and dyslipidemia in resource-limited settings, these factors play a significant role in exacerbating the prevalence and progression of pyoderma. Poor sanitation, limited access to healthcare, and unhealthy lifestyle choices further increase the risk of infections, including pyoderma, in these regions^28^. To effectively mitigate the burden of pyoderma, holistic strategies must be devised encompassing enhancements in socioeconomic conditions within low SDI areas, bolstering basic sanitation infrastructure, augmenting the scope and efficacy of public health interventions, while ensuring equitable access to healthcare services for socially marginalized populations in high SDI regions. These multifaceted interventions not only directly alleviate the burden of pyoderma but also contribute to the amelioration of health disparities and the promotion of global health equity.

Regarding age and gender distribution, our study discerns a heightened prevalence of pyoderma among children under 5 years of age, aligning with previous investigations^7,27^. Although the mortality rate remains low, the burden of DALYs remains disproportionately high, particularly evident in regions characterized by intermediate, low-intermediate, and low SDIs. A recent review of childhood skin diseases in tropical and subtropical regions of the developing world underscores pyoderma’s prevalence, typically ranging from 5 to 10%, attributed to a blend of factors encompassing immature immune systems and suboptimal personal hygiene practices in children^1,10,29^. The Guidelines for Integrated Management of Childhood Illnesses (IMCI), developed by the World Health Organization (WHO) and the United Nations Children’s Fund (UNICEF), serves as a pivotal strategy for identifying and treating illnesses in children under 5 years of age in low- and middle-income countries^30^. Despite pyoderma’s non-fatal nature, its protracted disease duration and adverse impact on quality of life necessitate a robust focus on prevention and early intervention^12^. Hence, bolstering parental awareness and education, fostering personal hygiene practices among children, conducting routine child health assessments, and channeling enhanced healthcare resources toward pediatric health and well-being emerge as critical tenets in pyoderma prevention and control efforts^13^.

Furthermore, with advancing age, middle-aged and elderly individuals afflicted with pyoderma exhibit heightened ASMR and ASDR, attributable to the prevalence of underlying comorbidities such as diabetes mellitus, hypertension, and hyperlipidemia, alongside age-related immune decline^5,8^. Consequently, for this demographic cohort, maintaining salubrious lifestyle habits, managing chronic ailments, prioritizing personal hygiene, and undergoing regular medical surveillance constitute imperative measures to forestall pyoderma incidence and mitigate its deleterious sequelae. Given the age-related disparities in pyoderma burden, there is an urgent need for age-specific healthcare interventions. For children under 5 years old, who are most affected by pyoderma in terms of incidence, increased healthcare resources should be directed toward pediatric care, including prevention, early intervention, and the management of skin diseases. Moreover, the rising ASDR and ASMR in older populations, particularly those aged 45 and above, necessitate targeted healthcare strategies that focus on managing comorbidities and enhancing the quality of care for the elderly. These findings reinforce the need for tailored healthcare policies and resource allocation that consider age-related disease burdens, particularly in regions with limited healthcare infrastructure.

The epidemiology of pyoderma is significantly influenced by population growth and ageing, particularly pronounced in regions characterized by middle, low-middle, and low SDI region, thereby necessitating targeted prevention and intervention strategies tailored to these contexts. Acknowledging the regional heterogeneity is imperative in crafting nuanced policies and measures aimed at mitigating the burden of pyoderma. To this end, we conducted frontier analyses leveraging ASIR, ASMR, ASDR, and SDI metrics to identify countries exhibiting exceptional health outcomes despite resource constraints, serving as exemplars for others. Surprisingly commendable performances were observed in select low SDI countries, whereas certain high SDI nations exhibited suboptimal outcomes. Future endeavors should delve into deciphering the determinants of success in frontier countries while elucidating the root causes of pyoderma-related health disparities, thereby fostering global pyoderma improvements and narrowing inter-country health inequalities. Projections based on the Bayesian Age-Period-Cohort (BAPC) model delineate a modest uptick in global pyoderma morbidity, mortality, and DALY rates over the ensuing decade, underscoring the persistent public health exigency of pyoderma warranting sustained attention and concerted response efforts. In light of this trajectory, heightened surveillance and prevention measures for pyoderma, judicious resource allocation, and context-specific policy formulations emerge as pivotal strategies to confront the evolving challenges posed by pyoderma^31^.

Through a comprehensive analysis of the longitudinal global trends in the incidence, mortality, and DALYs of pyoderma in relation to the level of socio-economic development, age, and gender, we can observe the crucial role of effective implementation of public health policies and improvement of basic sanitation in reducing the burden of pyoderma. These findings not only provide important epidemiological evidence for disease monitoring and prevention but also offer guidance for future public health interventions and resource allocation. The findings of this study are of great significance for the development of effective public health strategies and policies. Given the significant burden of pyoderma in low SDI countries, we highlight the importance of several public health interventions. First, we emphasize the need for awareness campaigns aimed at raising public awareness of pyoderma, particularly in regions with limited resources. These campaigns should focus on promoting personal hygiene and educating the public on the importance of early detection. Second, we propose the implementation of early detection initiatives, which would ensure that individuals at risk, especially children and the elderly, are diagnosed and treated promptly. This would help prevent long-term complications and reduce the disease burden. Finally, we recommend improving access to healthcare, particularly in at-risk populations. Strengthening primary healthcare services and expanding their reach in resource-poor settings is essential to reducing the impact of pyoderma.

The distribution of pyoderma globally and its association with socio-economic factors emphasize the need to improve sanitation conditions and enhance accessibility to public health services in low SDI regions, as well as to address antibiotic treatment for pyoderma in high SDI regions. Many European countries have established primary care databases, such as the Information System for the Development of Research in Primary Care in Catalonia or the Health Search Database in Italy^32–34^. These databases can be used to examine national trends in infectious disease cases and prescriptions, providing a strong potential for an international infectious disease surveillance network. The differential impacts of gender and age on the burden of pyoderma reveal the need for targeted interventions. For the high-risk pediatric population, increased investment in basic sanitation infrastructure and public health education is necessary. Additionally, attention should be strengthened for the elderly population, particularly in low SDI regions, and gender-specific health strategies should be considered. Lastly, this study underscores the importance of long-term monitoring and research to better understand the epidemiological trends of pyoderma and its underlying social determinants. This not only helps assess the effectiveness of implemented policies but also provides a scientific basis for future public health interventions.

While this study offers valuable insights into the global burden of pyoderma, it is important to acknowledge several limitations. Firstly, constraints in data sources may have led to underreporting of pyoderma cases or variations in diagnostic accuracy across countries and regions, potentially impacting the precision of incidence, mortality, and DALYs estimates. Secondly, despite efforts to delve into the epidemiological characteristics of pyoderma through age and sex stratification, other influential factors such as environmental shifts, socio-cultural disparities, and disparities in healthcare accessibility remained inadequately addressed. Additionally, reliance on public health databases and literature reviews might not fully encapsulate the latest research advancements and field study data.

Future research endeavors should prioritize the following areas: Firstly, expanding the geographical ambit and adopting more robust data collection and analysis methodologies to address data gaps, particularly enhancing the capacity for reporting and diagnosing pyoderma cases in low SDI countries, and leveraging AI and big data analytics to enhance data accuracy and efficiency. Secondly, delving deeper into the gender, age, and socio-economic dynamics impacting pyoderma morbidity and mortality, encompassing variables such as socio-economic status, educational attainment, living conditions, and hygiene practices. Lastly, spotlighting the efficacy and cost-effectiveness of pyoderma control measures, particularly in resource-constrained settings, and evaluating diverse public health interventions to underpin evidence-based pyoderma control strategies. These research trajectories hold promise for fostering a deeper comprehension and furnishing effective interventions aimed at alleviating the global burden of pyoderma.

## Conclusion

This study offers a comprehensive analysis of the global burden of pyoderma spanning from 1990 to 2019, unveiling substantial disparities in ASIR, ASMR, ASDR and disease burden across countries, regions, and socio-economic strata. The findings underscore the heightened pyoderma burden in regions characterized by low SDI regions, while delineating variations in burden distribution by gender, age, and the persistence of health inequities. In light of these revelations, forthcoming public health agendas and interventions must prioritize these regions and demographic cohorts, employing targeted measures to mitigate the pyoderma burden. Simultaneously, future research endeavors ought to surmount data limitations, delve deeper into the influence of socio-economic and cultural determinants, and evaluate the efficacy of preventive and therapeutic interventions to further ameliorate health disparities. These endeavors furnish a crucial foundation for the prospective development of more efficacious public health strategies and interventions aimed at alleviating the global burden of pyoderma.

## Electronic supplementary material

Below is the link to the electronic supplementary material.

Supplementary Material 1

## Data Availability

The datasets generated and analysed during the current study are available in the GBD 2019 database repository, accessible through the GBD results tool on the Institute for Health Metrics and Evaluation (IHME) website (http://ghdx.healthdata.org/).
